# The association of apolipoprotein B with chronic kidney disease in the Chinese population

**DOI:** 10.3389/fendo.2023.1083614

**Published:** 2023-04-12

**Authors:** Yu Xu, Bo Liu, Lijin Lin, Fang Lei, Tao Sun, Xingyuan Zhang, Xiaohui Song, Xuewei Huang, Qiang Zeng, Jingjing Cai, Zhifang Wang, Hongliang Li

**Affiliations:** ^1^ Department of Cardiology, Renmin Hospital of Wuhan University, Wuhan, China; ^2^ Department of Anesthesiology, Changsha Central Hospital, Changsha, China; ^3^ Institute of Model Animal, Wuhan University, Wuhan, China; ^4^ Medical Science Research Center, Zhongnan Hospital of Wuhan University, Wuhan, China; ^5^ School of Basic Medical Science, Wuhan University, Wuhan, China; ^6^ Department of Cardiology, The Third Xiangya Hospital, Central South University, Changsha, China; ^7^ Health Management Institute, The Second Medical Center & National Clinical Research Center for Geriatric Diseases, Chinese PLA General Hospital, Beijing, China; ^8^ Department of Nephrology Huanggang Central Hospital of Yangtze University, Huanggang, China; ^9^ Huanggang Institute of Translational Medicine, Huanggang Central Hospital of Yangtze University, Huanggang, China

**Keywords:** apolipoprotein B, chronic kidney disease, estimated glomerular filtration rate, dyslipidemia, atherosclerosis

## Abstract

**Background:**

Whether serum apolipoprotein B (ApoB) is a risk factor for the development of Chronic kidney disease (CKD) has not been fully established in the general population. Therefore, our study evaluated the correlation between serum ApoB level and CKD to look for an alternative approach for CKD prevention and treatment in the general population.

**Methods:**

There were 146,533 participants in this cross-sectional study. 3,325 participants with more than 2 measurements were enrolled in the retrospective longitudinal study with at least a 3-year follow-up. ApoB was measured by the immunoturbidimetric method in 6 centers. Our study defined CKD as an estimated glomerular filtration rate (eGFR) < 90 mL/min/1.73 m^2^. The Spearman rank correlation analysis and the Random Forest algorithm were applied to rank the importance of variables determining the levels of eGFR. We used the logistic regression model to explain the correlation between serum ApoB and CKD. We used the Cox model to detect the correlation between baseline serum ApoB and the subsequent occurrence of CKD.

**Results:**

Based on a cross-sectional study, 66.5% of the participants were males, with a median age of 49 (interquartile range [IQR] 43-55). Compared to the non-CKD group, the CKD group has higher levels of lipid profile and fasting glucose as well as the proportion of hypertension and hyperuricemia. The Spearman rank correlation analysis and the Random Forest algorithm revealed that ApoB has the highest correlation with eGFR decline among lipid profiles. The logistic regression analysis revealed that ApoB had a positive correlation with the prevalence of CKD after fully controlling confounders (odds ratio [OR], 1.07; 95% confidence interval [CI]: 1.02-1.11). Moreover, baseline ApoB level was correlated with a new-onset CKD in the longitudinal cohort after full adjustment for confounding factors (hazard ratio [HR], 1.61; 95% CI: 1.02-2.54). The correlation between ApoB level and the new-onset CKD was consistently observed in all sensitivity analyses.

**Conclusion:**

Serum ApoB had the strongest correlation with CKD among all lipid variables. Moreover, high serum ApoB levels might precede the occurrence of CKD, suggesting that monitoring and reducing serum ApoB levels may provide an alternative method to prevent and treat CKD.

## Background

As a major global public health problem, now chronic kidney disease (CKD), with a worldwide prevalence of 9.1% (1), is rising gradually and particularly in Asian countries with aging populations like China (2). CKD is associated with high morbidity and mortality, and is expected to become the fifth leading cause of death globally by 2040 (3). CKD has a serious impact on life quality and family economic status. The average per-patient to health care costs when the disease progresses to end-stage renal disease ranges from $20,110 to $100,593, without accounting for societal costs and productivity loss (4). Metabolic abnormalities are risk factors for CKD progression (5). Dyslipidemia is one of the most important components of metabolic abnormalities (6), which may contribute to renal lipid disturbances or aggravate glomerular and tubulointerstitial disease through the combination of inflammation and oxidative stress (7). Therefore, changes in lipid levels are important indicators reflecting the decline of renal function. Apolipoprotein B (ApoB) is a specific lipoprotein composition of serum lipids and has not received much attention in the previous studies on the relation of dyslipidemia and CKD compared with the traditional lipid parameters. Recent papers have found that serum ApoB level is a better biomarker for CVD of the risk diagnosis, prognosis (8, 9), and lipid-lowering therapy than traditional lipid factors. Whether ApoB plays a character in the development of CKD, previous studies are few and the results are inconclusive.

At present, lipid-lowering therapy by reducing the production of ApoB is mainly used in patients with atherosclerosis (10, 11). For example, mipomersen is the first Food and Drug Administration approved antisense ApoB synthesis inhibitor for patients who tolerate statin therapy and are at high CVD risk (12, 13). In the study of clinical trials, dose-dependent reductions were produced by mipomersen in different types of ApoB-containing lipoproteins, including lipoprotein (a) [Lp(a)] and low-density lipoprotein-cholesterol (LDL-C) (14). If ApoB proves to be a risk factor for CKD, mipomersen may be an alternative strategy for alleviating such metabolic risk-associated impairment in kidney function.

The current study aimed to examine the correlation between serum ApoB levels and CKD both on cross-sectional and longitudinal based on Chinese health check-up centers. If this hypothesis is established, it will provide an alternative approach to CKD prevention and treatment.

## Methods

### Study population

**Figure 1:**
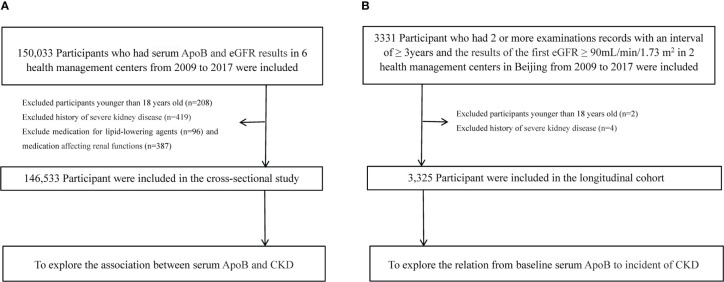
Flow chart of participants. **(A)** Flow chart of participants in the cross-sectional analysis. **(B)** Flow chart of participants in the longitudinal analysis.

150,033 individuals had tested for serum ApoB and creatinine in six health management centers screened in this study from January 2009 to December 2017. All the participants who entered this study followed voluntary bases. Individuals who were less than 18 years old (n=208), used lipid-lowering agents (n=96), drugs that affect kidney function (n=387), and with a history of severe kidney disease (renal carcinoma, nephrectomy, kidney transplant, nephrotuberculosis) (n=419) were excluded. The severe kidney disease may be accompanied by changes in nutritional status, which may be a potential confounding factor affecting the measurement of serum creatinine and leading to inaccurate estimates of renal function. There were 146,533 participants eligible for the cross-sectional study (**Figure 1A**). Individuals with estimated glomerular filtration rate (eGFR) ≥ 90 mL/min/1.73 m^2^ at the initial test and had at least two examinations for serum ApoB and creatinine with an interval of over 3 years were selected for the retrospective longitudinal cohort. There were 3,331 participants from 2 health management centers in Beijing from 2009 to 2017. After excluding 2 participants younger than 18 years old and 4 participants with a history of severe kidney disease. Finally, 3,325 participants were included to calculate the correlation between serum ApoB level and the incidence of CKD (**Figure 1B**). The study was approved by the central ethics board of Renmin Hospital. Individual identification data was removed, and only anonymous information was kept during the study. The ethics committees from each hospital waived patient informed consent.

### Anthropometric and laboratory data

All the individuals underwent clinical examinations and anthropometric measurements by experienced medical teams in the health check-up centers. The height (cm) was measured with shoes off (nearest 0.1 cm), and the weight (kg) was measured without a heavy coat (nearest 0.1 kg) (15). The automatic electronic sphygmomanometer was used for the blood pressure (mmHg) measurements after resting for 5-10 minutes (16). The blood sample was taken from the anterior axillary vein on an empty stomach. Biochemical tests and blood routine tests were detected by an automatic biochemical analyzer in a fasting condition. Two hours after the blood sample was collected. Test methods and procedures are unified laboratory standard protocols and guidelines (17). The concentrations of blood glucose, white blood cell (WBC), hemoglobin (HGB), alanine aminotransferase (ALT), triglyceride (TG), LDL-C, high-density lipoprotein-cholesterol (HDL-C), total cholesterol (TC), serum creatinine (Scr), blood uric acid (SUA), and blood urea nitrogen (BUN) were detected by the automatic biochemical analyzer. The six health management centers included in this study used the same method, namely the Jaffe method, to detect Scr (18). Apolipoprotein A-I (ApoA-1) and ApoB were detected by polyethylene glycol-enhanced immunoturbidimetry (19). The medical history and medication were collected based on self-report records during the health exam. Body mass index (BMI) was calculated as weight divided by height square (kg/m^2^). Past medical history of diseases and medications was obtained based on the self-reported history in the physical examination record.

### Diagnostic criteria

The estimated GFR was calculated by the Modification of Diet in the Renal Disease equation for Chinese patients (2006) (20). The eGFR < 90 mL/min/1.73 m^2^ (CKD stages 2-5) was defined as impaired kidney function based on the criteria in Kidney Disease Improving Global Outcomes (KDIGO) (21). Elevated ApoB level was described as a serum ApoB level above 1.1 g/L, according to the Chinese Guidelines for the Prevention and Treatment of Dyslipidemia in adults (19). Systolic blood pressure (SBP) ≥ 140 mmHg, or/and diastolic blood pressure (DBP) ≥ 90 mmHg, or a history of hypertension were identified as hypertension (22). Fasting plasma glucose ≥7.0 mmol/L, blood glucose 2 hours postprandial ≥ 11.1 mmol/L, history of hypoglycemic drugs, or history of diabetes were identified as diabetes (23). We defined hyperuricemia as an SUA > 360 μmol/L (6.0 mg/dL) and SUA > 420 μmol/L (7.0 mg/dL) for females and males, respectively (24). We defined hypohemia as an HGB < 110 g/L in females and < 120 g/L in males which is based on the diagnostics definitions (25).

### Statistical analysis

Categorical variables were represented as frequencies and percentages. Continuous variables were expressed as the median and interquartile range (IQR). Student’s t-test was used for testing the difference in the two groups for variables with normal distribution, and the Kruskal-Wallis test was applied to two groups with skewed distribution. Fisher’s exact test or χ^2^ test was used for comparisons in categorical variables. The variables with a miss data rate of < 30% were complemented using Miss Forest (26). We used the Spearman rank correlation analysis and the Random Forest algorithm to select the important variables to determine the eGFR levels. The common method - logistic regression was selected for detecting the correlation between serum ApoB level and CKD prevalence. We determined the correlation between baseline ApoB level and the subsequent occurrence of CKD under the Cox proportional hazards regression model. The mixed-effects Cox regression models was applied in sensitivity analysis II (27).Two-sided *P* < 0.05 was considered statistically significant. We used SPSS Statistics (version 25.0, IBM, Armonk, NY, USA) and R-4.0.2 (R Foundation for Statistical Computing, Vienna, Austria) for data analysis.

### Sensitivity analysis

We further conducted two sensitivity analyses of the correlation between baseline ApoB level and the subsequent occurrence of CKD in the retrospective cohort for quality control. In sensitivity analysis I, we validated the results by increasing the duration of the follow-up. There were 1,896 participants with two or more health check-up records with an interval of ≥ 4 years between examinations. In sensitivity analysis II, we used the mixed-effects Cox regression models (medical center as a random effect) to assess the relationship between baseline ApoB level and the new-onset CKD. E-value analysis to address potential unmeasured confounding effects in the Cox model to assess the robustness of the association between ApoB and CKD occurrence (28).

## Results

### Participants’ characteristics of the cross-sectional population

The study included data from 146,533 participants (median age, 49 years; IQR, 43-55 years), and 66.5% were males. The median level of ApoB was 0.91 (IQR, 0.77-1.08) g/L, the median Scr level was 69.00 (IQR, 59.00-78.20) μmol/L, the median SUA level was 336.00 (IQR, 274.00-398.00) μmol/L, and the median BUN level was 5.00 (IQR, 4.20-5.83) mmol/L. 22.5% had hypertension, 14.3% had hyperuricemia, and 13.3% suffered from diabetes. A comparison of participants across eGFR normal and abnormal groups revealed that those individuals with low levels of eGFR had generally higher levels of age (53 years [IQR, 47-62] versus 48 years [IQR, 42-54], *P* < 0.001), BMI (25.56 kg/m^2^ [IQR, 23.65-27.53] versus 25.01 kg/m^2^ [IQR, 22.79-27.29], *P* < 0.001), SBP (126 mmHg [IQR, 115-140] versus 121 mmHg [IQR, 110-134], *P* < 0.001) and ApoB (0.95 g/L [IQR, 0.80-1.12] versus 0.91 g/L [IQR, 0.76-1.07], *P* < 0.001). Detailed characteristics are provided in **Table 1**.

**Table 1:** Baseline characteristics of the cross-sectional population.

Characteristic	Overall	eGFR ≥ 90 ml/min/1.73 m^2^	eGFR< 90 ml/min/1.73 m^2^	*P* value*
	(N=146533)	(N=127447)	(N=19086)	
Sex, male, n(%)	97583 (66.5)	81893 (64.3)	15690 (82.2)	<0.001
Age, median[IQR]years	49 [43-55]	48 [42-54]	53 [47-62]	<0.001
BMI (kg/m^2^, median[IQR])	25.09 [22.89-27.34]	25.01 [22.79-27.29]	25.56 [23.65-27.53]	<0.001
SBP (mmHg, median[IQR])	122 [110-135]	121 [110-134]	126 [115-140]	<0.001
DBP (mmHg, median[IQR])	78 [70-85]	78 [70-85]	80 [72-88]	<0.001
Serum glucose (mmol/L, median[IQR])	5.30 [4.92-5.84]	5.30 [4.91-5.84]	5.34 [4.96-5.83]	<0.001
TG (mmol/L, median[IQR])	1.43 [0.98-2.12]	1.41 [0.97-2.11]	1.55 [1.10-2.21]	<0.001
TC (mmol/L, median[IQR])	4.83 [4.23-5.48]	4.81 [4.22-5.46]	4.94 [4.32-5.61]	<0.001
LDL-C (mmol/L, median[IQR])	2.99 [2.46-3.53]	2.98 [2.46-3.52]	3.05 [2.52-3.59]	<0.001
HDL-C (mmol/L, median[IQR])	1.22 [1.02-1.47]	1.23 [1.03-1.48]	1.19 [1.01-1.42]	<0.001
ApoA-I (g/L, median[IQR])	1.30 [1.14-1.48]	1.30 [1.15-1.48]	1.27 [1.12-1.45]	<0.001
ApoB (g/L, median[IQR])	0.91 [0.77-1.08]	0.91 [0.76-1.07]	0.95 [0.80-1.12]	<0.001
HGB (g/L, median[IQR])	147.00 [135.00-157.00]	147.00 [134.00-157.00]	150.00 [139.80-159.00]	<0.001
WBC (10^9/L, median[IQR])	5.93 [5.03-7.00]	5.90 [5.00-7.00]	6.04 [5.16-7.10]	<0.001
ALT (U/L, median[IQR])	20.00 [14.30-29.60]	20.00 [14.10-29.70]	20.60 [15.00-29.00]	<0.001
Estimated GFR (ml/min/1.73 m^2^, median[IQR])	112.06 [98.23-128.88]	115.88 [103.63-131.76]	82.75 [76.23-86.83]	<0.001
Scr (μmol/L, median[IQR])	69.00 [59.00-78.20]	67.00 [57.00-75.00]	90.50 [86.00-96.30]	<0.001
SUA (μmol/L, median[IQR])	336.00 [274.00-398.00]	329.00 [268.10-391.00]	380.30 [325.00-440.50]	<0.001
BUN (mmol/L, median[IQR])	5.00 [4.20-5.83]	4.90 [4.11-5.7]	5.70 [4.80-6.68]	<0.001
Hypertension, n(%)	33067 (22.5)	27441 (24.7)	5626 (34.8)	<0.001
Diabetes, n(%)	19512 (13.3)	16954 (13.4)	2558 (13.5)	0.600
Hyperuricemia, n(%)	21092 (14.3)	15864 (12.4)	5228 (27.4)	<0.001

IQR, interquartile range; BMI, body mass index; SBP, systolic blood pressure; DBP, diastolic blood pressure; TG, triglyceride; TC, total cholesterol; LDL-C, low-density lipoprotein-cholesterol; HDL-C, high-density lipoprotein-cholesterol; ApoA-I, apolipoprotein A-I; ApoB, apolipoprotein B; HGB, hemoglobin; WBC, white blood cell; ALT, alanine aminotransferase; eGFR, estimated glomerular filtration rate; Scr, serum creatinine; SUA, Serum uric acid; BUN, blood urea nitrogen.

*P values were calculated by the Kruskal-Wallis test for continuous variables, as well as the chi-square test or Fisher’s exact test for categorical variables.

### Association between lipid variables and CKD

**Supplementary Table 1** shows the correlation matrix of serum lipid variables with CKD (eGFR < 90 ml/min/1.73 m^2^). Spearman rank correlation analysis shows that ApoB was directly proportional to CKD (r = 0.063, *P* < 0.001). Besides ApoB, TG (r = 0.055, *p* < 0.001), TC (r = 0.042, *P* < 0.001), and LDL-C (r= 0.027, *P* < 0.001) were directly proportional to CKD, whereas HDL-C (r = −0.039, *P* < 0.001) and AopA-I (r = −0.037, *P* < 0.001) was negatively associated with CKD prevalence. In the screening of important variables of random forest, ApoB (Mean Decrease Accuracy, 118.455) was considered the most important indicator among all lipid variables.

### Association between ApoB and CKD prevalence in the cross-sectional population

This study applied logistic regression analysis to identify the correlation between serum ApoB level and CKD prevalence. In the crude model, there is a strong connection between ApoB and CKD (odds ratio [OR], 1.37; 95% confidence interval [CI]: 1.32-1.42) (*P* < 0.001). After adjusting for sex, age, BMI, hypertension, diabetes, hyperuricemia, TG, TC, WBC, HGB, alcohol use, and smoke status, serum ApoB level was still significantly positively correlated with CKD prevalence. The OR value was 1.07 (95% CI: 1.02-1.11) (*P* = 0.006) (**Table 2**).

**Table 2:** Association between ApoB and the prevalence of CKD in the cross-section population.

Model	OR(95%CI)		*P* value*
	ApoB(g/L) ≤ 1.1	ApoB(g/L) > 1.1	
Crude	Ref	1.37 (1.32,1.42)	< 0.001
Model1	Ref	1.30 (1.26,1.35)	< 0.001
Model2	Ref	1.07 (1.02,1.11)	0.006

OR, odds ratio; CI, confidence interval; ApoB, apolipoprotein B; CKD, chronic kidney disease; BMI, body mass index; TG, triglyceride; TC, total cholesterol; HGB, hemoglobin; WBC, white blood cell.

Model1, adjusted for age, sex and BMI.

Model2, adjusted for age, sex, BMI, hypertension, diabetes, hyperuricemia, TG, TC, WBC, HGB, alcohol use and smoke status.

*P values were calculated based on logistic regression model.

### Baseline characteristics of longitudinal cohort

Longitudinal analysis was further conducted to verify the correlation between baseline ApoB and CKD incidence. A strong association between serum ApoB levels and CKD prevalence was found in cross-sectional analysis. In a three-year follow-up cohort of 3325 individuals with eGFR ≥ 90 ml/min/1.73 m^2^ at baseline, 185 individuals developed eGFR decline (eGFR < 90mL/min/1.73 m^2^). The population′s median age of the eGFR decline group was 50 (IQR, 45-55) years, older than those with the normal eGFR group (47 [IQR, 42-51] years). The participants that developed eGFR decline had larger proportions of males (87.60% versus 67.20%), hypertension (22.80% versus 18.20%), and hyperuricemia (19.50% versus 11.90%). Accordingly, the levels of BMI, SBP, DBP, WBC, ALT, LDL-C, TC, TG, Scr, SUA, and BUN were higher in the developed eGFR decline group. There is a higher proportion of participants with an elevated ApoB level developed eGFR decline. Detailed characteristics are provided in **Table 3**.

**Table 3:** Baseline characteristics of the longitudinal cohort.

Characteristic	Overall	eGFR ≥ 90 ml/min/1.73 m^2^	eGFR < 90 ml/min/1.73 m^2^	*P* value*
	(N=3325)	(N=3140)	(N=185)	
Sex, male, n(%)	2273 (68.30)	2111 (67.20)	162 (87.60)	<0.001
Age, median[IQR] years	47 [42-52]	47 [42-51]	50 [45-55]	<0.001
BMI (kg/m^2^, median[IQR])	25.30 [23.23-27.42]	25.27 [23.18-27.42]	25.55 [23.88-27.42]	0.043
SBP (mmHg, median[IQR])	119 [109-130]	119 [109-130]	120 [112-133]	0.006
DBP (mmHg, median[IQR])	78.00 [71.00-85.00]	77.00 [71.00-85.00]	80.00 [74.00-85.25]	0.004
Serum glucose (mmol/L, median[IQR])	5.35 [4.97-5.89]	5.36 [4.97-5.90]	5.32 [4.95-5.79]	0.753
TG (mmol/L, median[IQR])	1.53 [1.05-2.32]	1.52 [1.04-2.30]	1.71 [1.20-2.43]	0.054
TC (mmol/L, median[IQR])	4.85 [4.26-5.50]	4.84 [4.26-5.49]	5.02 [4.29-5.64]	0.214
LDL-C (mmol/L, median[IQR])	3.06 [2.51-3.60]	3.05 [2.51-3.60]	3.15 [2.53-3.76]	0.209
HDL-C (mmol/L, median[IQR])	1.17 [0.99-1.40]	1.17 [0.99-1.40]	1.14 [0.99-1.31]	0.120
ApoA-I (g/L, median[IQR])	1.31 [1.17-1.48]	1.31 [1.17-1.48]	1.29 [1.16-1.43]	0.240
ApoB (g/L, median[IQR])	0.89 [0.74-1.05]	0.89 [0.74-1.05]	0.90 [0.75-1.10]	0.124
HGB (g/L, median[IQR])	149 [137-158]	149 [136-158]	154 [145-160]	<0.001
WBC (10^9/L, median[IQR])	5.80 [4.95-6.84]	5.79 [4.94-6.83]	6.16 [5.27-7.08]	0.004
ALT (U/L, median[IQR])	21.50 [14.7-32.20]	21.40 [14.60-32.20]	23.20 [16.65-31.73]	0.147
Estimated GFR (ml/min/1.73 m^2^, median[IQR])	116.03 [104.69-130.82]	117.17 [106.27-131.89]	97.30 [93.30-103.71]	<0.001
Scr (μmol/L, median[IQR])	67.00 [58.00-75.00]	67.00 [57.10-74.60]	79.90 [73.00-83.10]	<0.001
SUA (μmol/L, median[IQR])	341.00 [277.30-400.50]	338.00 [275.00-399.00]	373.00 [339.00-430.00]	<0.001
BUN (mmol/L, median[IQR])	5.04 [4.30-5.90]	5.00 [4.30-5.90]	5.40 [4.60-6.18]	<0.001
Hypertension, n(%)	613 (18.40)	571 (18.20)	42 (22.80)	0.140
Diabetes, n(%)	439 (13.20)	417 (13.30)	22 (11.90)	0.670
Hyperuricemia, n(%)	410 (12.30)	374 (11.90)	36 (19.50)	<0.001

IQR, interquartile range; BMI, body mass index; SBP, systolic blood pressure; DBP, diastolic blood pressure; TG, triglyceride; TC, total cholesterol; LDL-C, low-density lipoprotein-cholesterol; HDL-C, high-density lipoprotein-cholesterol; ApoA-I, apolipoprotein A-I; ApoB, apolipoprotein B; HGB, hemoglobin; WBC, white blood cell; ALT, alanine aminotransferase; eGFR, estimated glomerular filtration rate; Scr, serum creatinine; SUA, Serum uric acid; BUN, blood urea nitrogen.

*P values were calculated by the Kruskal-Wallis test for continuous variables, as well as the chi-square test or Fisher’s exact test for categorical variables.

### Association between the baseline ApoB and incident of CKD in the longitudinal cohort

Individuals with increased baseline serum ApoB showed a greater risk of developing incident CKD during the three-year follow-up in the crude analysis, with a hazard ratio (HR) of 1.50 (95% CI: 1.07-2.11) (*P* = 0.019). After adjusting for age, sex, BMI, hypertension, diabetes, hyperuricemia, TG, TC, WBC, HGB, alcohol use, and smoke status, the baseline ApoB increase was still correlated with the occurrence of CKD (HR, 1.61; 95% CI: 1.02-2.54) (*P* = 0.042) (**Table 4**).

**Table 4:** Association between the baseline ApoB and incident of CKD in the longitudinal cohort.

Model	HR(95%CI)		*P* value*
	ApoB(g/L) ≤ 1.1	ApoB(g/L) > 1.1	
Crude	Ref	1.50 (1.07,2.11)	0.019
Model1	Ref	1.48 (1.05,2.08)	0.024
Model2	Ref	1.61 (1.02,2.54)	0.042

HR, hazard ratio; CI, confidence interval; ApoB, apolipoprotein B; CKD, chronic kidney disease; BMI, body mass index; TG, triglyceride; TC, total cholesterol; HGB, hemoglobin; WBC, white blood cell.

Model1, adjusted for age, sex and BMI.

Model2, adjusted for age, sex, BMI, hypertension, diabetes, hyperuricemia, TG, TC, WBC, HGB, alcohol use and smoke status.

*P values were calculated based on Cox regression model.

### Subgroup analysis

**Figure 2:**
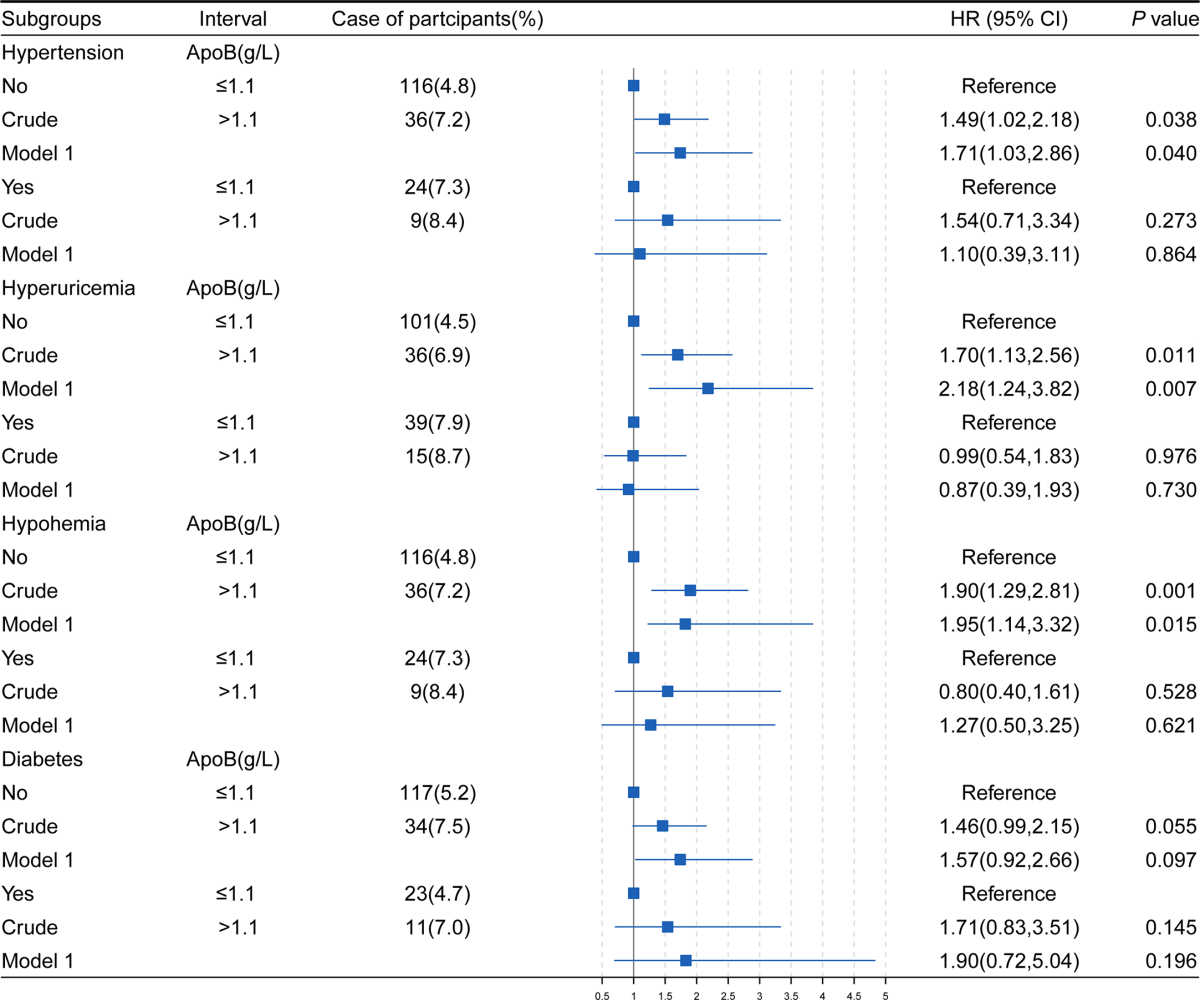
The association of ApoB and the development of CKD in study subjects stratifled by hypertension, hyperuricemia, hypohemia, diabetes. HR, hazard ratio; CI, confidence interval; BMI, body mass index; HGB, hemoglobin; ApoB, apolipoprotein B; TG, triglyceride; TC, total cholesterol; WBC, white blood cell. Model1, adjusted for age, sex, BMI, hypertension, diabetes, hyperuricemia, HGB, TG, TC, WBC, alcohol use and smoke status. *P values were calculated based on Cox regression.

The associations between baseline ApoB levels and the occurrence of CKD were analyzed in individuals with or without hypertension, diabetes, hyperuricemia, and hypohemia (**Figure 2**). In model 1, adjusting for confounders, the HRs were 1.71 (95% CI: 1.03-2.86) (*P* = 0.040) of the group without hypertension, 2.18 (95% CI: 1.24-3.82) (*P* = 0.007) of the group without hyperuricemia, 1.95 (95% CI: 1.14-3.32) (*P* = 0.015) of the group without hypohemia. However, in subgroups with hypertension, hyperuricemia and hypohemia, the associations between ApoB and CKD disappeared.

### Sensitivity analysis

In sensitivity analysis I, we validated the results by increasing the duration of the follow-up to 4 years. When adjusted for sex, age, BMI, hypertension, diabetes, hyperuricemia, TG, TC, WBC, HGB, alcohol use, and smoke status, ApoB at baseline remained significantly correlated with the CKD occurrence (HR, 1.76; 95% CI: 1.00-3.07) (*P* = 0.048) (**Table 5**). In sensitivity analysis II, the mixed-effects Cox regression model was applied. The association of the baseline ApoB and the development of CKD remained significant (HR, 1.62; 95% CI: 1.03-2.56) (*P* = 0.038) (**Table 6**). In the results of E-value analysis, we found that the estimated point of the primary endpoint in the Cox model was 2.60. Since this value is greater than the strong confounders, unmeasured confounders are unlikely to overcome the association between elevated ApoB and the occurrence of CKD

**Table 5:** Association between the baseline ApoB and incident of CKD in the 4 years longitudinal cohort.

Model	HR(95%CI)		*P* value*
	ApoB(g/L) ≤ 1.1	ApoB(g/L) >1.1	
Crude	Ref	1.83 (1.22, 2.75)	0.004
Model1	Ref	1.81 (1.20, 2.73)	0.004
Model2	Ref	1.76 (1.00, 3.07)	0.048

HR, hazard ratio; CI, confidence interval; ApoB, apolipoprotein B; CKD, chronic kidney disease; BMI, body mass index; TG, triglyceride; TC, total cholesterol; HGB, hemoglobin; WBC, white blood cell.

Model1, adjusted for age, sex and BMI.

Model2, adjusted for age, sex, BMI, hypertension, diabetes, hyperuricemia, TG, TC, WBC, HGB, alcohol use and smoke status.

*P values were calculated based on Cox regression model.

**Table 6:** Association between baseline ApoB and incident CKD by Mixed-effects Cox regression in the longitudinal cohort.

Model	HR (95%CI)		*P* value*
	ApoB(g/L) ≤ 1.1	ApoB(g/L) > 1.1	
Crude	Ref	1.51 (1.07,2.12)	0.018
Model1	Ref	1.49 (1.06,2.09)	0.022
Model2	Ref	1.62 (1.03,2.56)	0.038

Multivariable HR, Multivariable hazard ratio; CI, confidence interval; ApoB, apolipoprotein B; CKD, chronic kidney disease; BMI, body mass index; TG, triglyceride; TC, total cholesterol; HGB, hemoglobin; WBC, white blood cell.

Model1, adjusted for age, sex and BMI.

Model2, adjusted for age, sex, BMI, hypertension, diabetes, hyperuricemia, TG, TC, WBC, HGB, alcohol use, smoke status and medical center as random effect.

*P values were calculated based on Mixed-effects Cox regression model.

## Discussion

Our study identified the positive association between serum ApoB level and CKD both in the cross-sectional study and the retrospective cohort. As far as we know, the current study is the first in China to elucidate the correlation based on a large data set of 146,533 individuals. We found that ApoB outperformed other lipid characteristics with the highest correlation coefficients with CKD, and the increase of ApoB has a positive correlation with CKD prevalence after fully adjusting for covariates in the cross-sectional population. We also observed a positive correlation between high serum ApoB levels and new-onset eGFR decline in the longitudinal cohort. In our subgroup analyses, higher ApoB levels were correlated with the incidence of CKD in people with non-hypertension, non-hyperuricemic, and non-anemic after adjustment for confounders.

Dyslipidemia is known to be an independent risk factor for CKD. ApoB is a special apolipoprotein of lipid profile (29, 30). In this study, we first found that the serum ApoB had a relatively stronger correlation with CKD compared with other variables in lipids. In the cross-sectional study, serum ApoB increases were notably correlated with the prevalence of CKD stages 2-5. Our results are consistent with previous findings based on the Chinese population, showing a great positive relationship between ApoB and the stages of CKD in cross-section (31). The other cross-section study by Mazidi et al. is main finding that individuals with high ApoB levels is more likely to develop CKD (eGFR < 60 ml/min/1.73 m^2^) when LDL-C and ApoB are inconsistent, even after adjusting for a range of confounding variables (32). Ethnic differences may influence the association between ApoB and CKD. As shown in earlier cross-sectional research relied on the National Health and Nutrition Examination Survey (NHANES) III, ApoB was not associated with stages 3 to 5 of CKD (eGFR < 60 ml/min/1.73 m^2^) after adjusting for confounders (30). The high ApoB level at baseline predicted subsequent CKD development in this longitudinal cohort study. Consistent with our findings, in a study of Korean men followed for 5 years, higher serum ApoB and the ratio of ApoB to ApoA-I values were associated with lower level eGFR at baseline and a higher risk of subsequent eGFR reduction (eGFR < 60 ml/min/1.73 m^2^) (33). However, a retrospective longitudinal analysis of 10,288 subjects with a mean follow-up of 42.2-70.8 months showed that LDL-C/ApoB and HDL-C/ApoA-1 ratios, but not ApoB concentration, independently predicted an increased risk of developing CKD(eGFR < 60 ml/min/1.73 m^2^). The differences in cohort results may be due to the differences in sample size and follow-up time (34). Different from conventional blood tests, nuclear magnetic resonance spectroscopy was used to quantify plasma levels of lipoprotein particles and their lipid components and low eGFR was also found to be significantly correlated with high levels of ApoB and the count of lipoprotein particles that contain most of the ApoB in a prospective study in Mexico (35).

A comprehensive assessment of the association between decreased eGFR and lipid variables in Kazakh hypertensives reported that serum ApoB level is significantly negatively associated with the early pre-CKD condition, and the final model was still significantly fitted even after adjustment of confounding factors, including diet, age, quality score and income (36). Hence, for patients with different comorbidities, the associations of increased serum ApoB level transitions with the development of CKD, and whether comorbidities can modify the effect are worth exploring. Therefore, we analyze the relationship between ApoB and eGFR decline in participants with diabetes, hypertension, dyslipidemia, and hypohemia. We have noticed the weakened association between ApoB and CKD in patients with hypertensive, hyperuricemia and hypohemia. It is known that hypertensive, hyperuricemia, hypohemia, and diabetes are strong risk factors for the initiation and development of CKD (37–40). The association between ApoB and CKD may be masked in patients with such diseases. Our study provided a hypothesis that ApoB may be an independent risk factor for CKD, especially in populations with hypertensive, hyperuricemia and hypohemia. A perspective study is warranted to confirm the causal relationship between ApoB and CKD in the general population and in patients with comorbidities.

CKD is a slowly progressing disease exposed to multiple risk factors. Dyslipidemia is one of the important propathogenic factors. ApoB, as an important component of the lipid profile, may affect the development and progression of CKD through the following mechanisms. High levels of ApoB-containing lipoproteins may initiate and promote atherosclerosis. When the plasma LDL-C and very low-density lipoprotein increase, the proportion of lipoprotein particles that contain most of the ApoB entering the arterial wall increases, and the fraction that cannot diffuse back into the circulation binds to the arterial wall proteoglycans (41, 42). The interaction between proteoglycans and ApoB motivates the accumulation of lipid particles in the subendothelium, which can accelerate oxidation and inflammation in the vascular wall (43, 44). With high concentrations of ApoB, glomerular endothelial cells and renal vessels may undergo a high level of oxidation stress and inflammation, and arteriosclerosis occurs in small and medium vessels, leading to decreased eGFR and CKD progression (45, 46). The mechanisms underlying the interaction between apolipoproteins, dyslipidemia, and CKD are complex.

Currently, the reagent inhibits the production of ApoB and is mainly used for treating atherosclerosis. It was found that the ApoB-100 peptide P210 vaccine significantly attenuated aortic atherosclerosis in a humanized mouse model (47). The decrease of dose-dependent in apolipoproteins, such as Lp(a), and LDL-C, were observed after the administration of mipomersen in clinical trials (13). If ApoB proves to be a risk factor for CKD, mipomersen may be an alternative strategy for alleviating such metabolic risk-associated impairment in kidney function.

### Limitations

Some limitations exist in our study. First, due to the inherent bias of retrospective design, even if we use several statistical models to adjust for potential bias and perform sensitivity analyses to show that overall unmeasured confounders are unlikely to undermine our main conclusion, there will still be unforeseen confounders that could potentially alter the extent to which ApoB affects the occurrence of CKD. The relationship between serum ApoB and the incidence of CKD needs to be prospectively validated. Second, the population of this study was based on some community health examinations in China rather than random sampling, and there may be participant selection bias in this study. Third, using serum creatinine to estimate GFR, rather than direct measurement of kidney function, may overestimate or underestimate the actual GFR.

## Conclusion

In summary, serum ApoB level has the strongest correlation with CKD among all lipid variables in the Chinese population. Moreover, the increase of serum ApoB level might precede the occurrence of CKD, suggesting that monitoring and reducing serum ApoB levels may provide an alternative approach for the prevention and treatment of CKD.

## Data availability statement

The datasets used and analyzed during the current study are available from the corresponding author upon reasonable request. Requests to access the datasets should be directed to HL, lihl@whu.edu.cn.

## Ethics statement

The studies involving human participants were reviewed and approved by the central ethics board of Renmin Hospital.

## Author contributions

YX and BL designed the study, collected and analyzed data, and drafted the manuscript. LL, FL, and TS performed the statistical analysis and interpreted data. XZ, XS, XH, and QZ assisted in data collection. JC performed critical revision of the manuscript for important intellectual content. ZW and HL conceived and supervised the study and made critical revisions to the manuscript for important intellectual content. All authors contributed to the article and approved the submitted version.
