# Supplementary material for: The association of apolipoprotein B with chronic kidney disease in the Chinese population
Source: Front Endocrinol (Lausanne). 2023 Apr 12;14:1083614. doi: 10.3389/fendo.2023.1083614 (PMC10132458; doi:10.3389/fendo.2023.1083614)
Supplement: Supplementary file 1 [file Table_1.docx]

Supplementary Table 1. Association between lipid variables and the prevalence of CKD in the cross-section population

	Spearman correlation		Random Forest
	coefficients	*P* value*	Mean Decrease Accuracy
ApoB (g/L)	0.063	<0.001	118.455
TG (mmol/L)	0.055	<0.001	114.377
TC (mmol/L)	0.042	<0.001	99.263
HDL-C (mmol/L)	-0.039	<0.001	118.205
ApoA-I (g/L)	-0.037	<0.001	79.378
LDL-C (mmol/L)	0.027	<0.001	101.313

Abbreviation: CKD, chronic kidney disease; ApoB, apolipoprotein B; TG, triglyceride; TC, total cholesterol; HDL-C, high density lipoprotein-cholesterol; lipoprotein; ApoA-I, apolipoproteins A-I; LDL-C, low density lipoprotein-cholesterol.

Data is expressed as Spearman's coefficient and Random forest variable importance score.

**P* value were calculated based on Spearman’s coefficient.
